# The DNA repair protein ATM as a target in autism spectrum disorder

**DOI:** 10.1172/jci.insight.133654

**Published:** 2021-02-08

**Authors:** Lara Pizzamiglio, Elisa Focchi, Clara Cambria, Luisa Ponzoni, Silvia Ferrara, Francesco Bifari, Genni Desiato, Nicoletta Landsberger, Luca Murru, Maria Passafaro, Mariaelvina Sala, Michela Matteoli, Elisabetta Menna, Flavia Antonucci

**Affiliations:** 1 Department of Medical Biotechnology and Translational Medicine (BIOMETRA), University of Milan, Milan, Italy.; 2 Institute of Neuroscience, IN-CNR, Milan, Italy.; 3 Humanitas Clinical and Research Center – IRCCS, Rozzano, Milan, Italy.

**Keywords:** Development, Neuroscience, Molecular pathology, Mouse models, Neurological disorders

## Abstract

Impairment of the GABAergic system has been reported in epilepsy, autism, attention deficit hyperactivity disorder, and schizophrenia. We recently demonstrated that ataxia telangiectasia mutated (ATM) directly shapes the development of the GABAergic system. Here, we show for the first time to our knowledge how the abnormal expression of ATM affects the pathological condition of autism. We exploited 2 different animal models of autism, the methyl CpG binding protein 2–null (*Mecp2^y/–^*) mouse model of Rett syndrome and mice prenatally exposed to valproic acid, and found increased ATM levels. Accordingly, treatment with the specific ATM kinase inhibitor KU55933 (KU) normalized molecular, functional, and behavioral defects in these mouse models, such as (a) delayed GABAergic development, (b) hippocampal hyperexcitability, (c) low cognitive performances, and (d) social impairments. Mechanistically, we demonstrate that KU administration to WT hippocampal neurons leads to (a) higher early growth response 4 activity on *Kcc2b* promoter, (b) increased expression of Mecp2, and (c) potentiated GABA transmission. These results provide evidence and molecular substrates for the pharmacological development of ATM inhibition in autism spectrum disorders.

## Graphical abstract

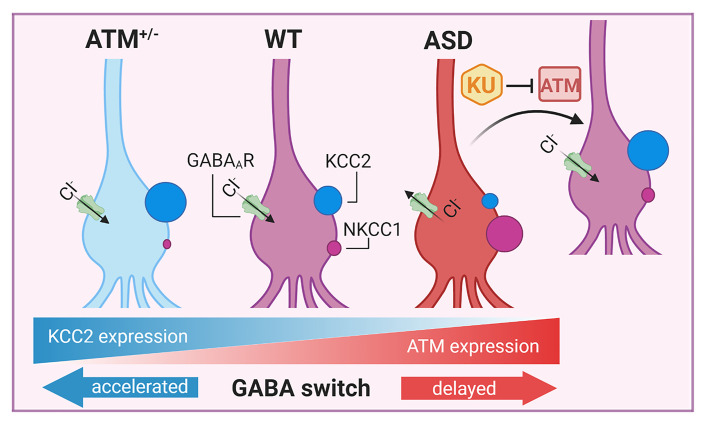

## Introduction

In several developmental diseases such as autism, Down syndrome, Dravet syndrome, Rett syndrome, perinatal neuroinflammation, and epilepsy, an altered GABA-mediated inhibition has been addressed (1–5), as well as an imbalanced excitatory/inhibitory ratio (E/I balance) (6–8). In fact, whereas glutamate mediates neuronal depolarization along life, at the early stages of neuronal development, GABA acts as an excitatory neurotransmitter rather than inhibitory (9–13), directly evoking action potentials and raising intracellular calcium levels (12, 14, 15). This excitatory action of GABA depends on the expression of the sodium-potassium-chloride cotransporter NKCC1, which maintains the high intracellular chloride concentration in immature neurons (9). Then, during neuronal development, it acquires its typical role of brake for neuronal activity through the important process called “excitatory-to-inhibitory switch of GABA” (also called GABA switch), which is directly related to the action of the potassium-chloride cotransporter KCC2. By extruding the chloride from neurons, KCC2 guarantees (a) a low intracellular ion concentration and (b) the inhibitory function of GABA upon the opening of GABA-A receptor. In line with these pivotal effects, impaired KCC2 expression or function associates to the generation of neurodevelopmental diseases, and accordingly, KCC2 enhancement is the basis of the new therapeutic strategy for these conditions (16).

Recently, we demonstrated that neurons expressing reduced levels of ataxia telangiectasia mutated (ATM), a protein involved in the DNA double strand breaks (DSBs) response, display increased KCC2 levels, premature GABA switch, and higher inhibitory tone (17). Moreover, several findings highlight ATM’s important involvement in fundamental neurobiological processes such as neuronal survival, cellular proliferation, and synaptic vesicle recycling (18–21). Also, a proper functioning of DSBs machinery (22) is necessary for proper development of cognitive abilities (23). Interestingly, a recent study identified 11 candidate single nucleotide polymorphisms and 6 genes contributing to attention deficit hyperactive disorder susceptibility, and among these the *ATM* gene is included (24). Also, in a Taiwanese Han population, a specific runs of homozygosity region associated with the language impairments of autism has been found on 11q22.3 chromosome, a region that contains the ATM gene (25). Thus, these genetic studies support a role of ATM in the etiology of developmental disorders.

Considering all the above, we hypothesized that the tuning of ATM kinase activity could be beneficial in neurodevelopmental pathologies and, to this scope, we investigated the effects of the ATM kinase inhibitor, KU55933 (KU). Here, we describe the capacity of KU to recover neuronal alterations found in methyl CpG binding protein 2–null (*Mecp2^y/–^*) neurons and in mice exposed to valproate (animals in utero exposed to VPA, VPA mice). Both are considered good models of autism spectrum disorders (ASD) and are characterized by a prolonged excitatory GABA action and hyperexcitability. Coherently, both models present, among the etiopathological alterations, reduced KCC2 levels (26–30). Also, we demonstrate that the higher KCC2 expression achieved by the inhibition of ATM kinase occurs through (a) the promotion of the activity of the early growth response 4 (Egr4) on *Kcc2b* promoter and (b) the increased expression of the epigenetic regulator Mecp2. Accordingly, both the mechanisms resulted in strengthening in *Atm* heterozygous neurons and tissues.

Thus, we highlight a potentially completely new and unexplored application of KU in neurodevelopmental disorders since up to now KU has been studied as an antiproliferative and radiosensitizer agent against tumors (31–34). It is a small molecule possibly able to generate unspecific actions, but here we also identify the lowest concentration able to guarantee both safety and effectiveness in neurons.

This study indicates that the inhibition of ATM kinase activity, achieved by KU, may revert functional features in autism by restoring the proper development of inhibition and by preventing the occurrence of deleterious effects linked to neuronal hyperexcitability driven by an excitatory GABA action.

## Results

### ATM expression is higher in hippocampal tissues of 2 animal models of autism.

**Figure 1:**
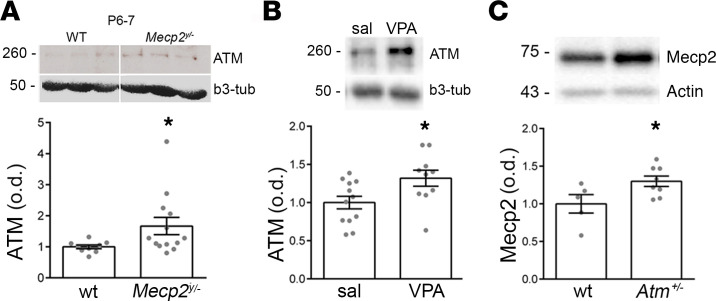
Increased ATM levels in animal models of autism. (**A** and **B**) ATM levels evaluated in WT (*n* = 10) vs. *Mecp2^y/–^* pups (*n* = 13), Mann-Whitney *U* test: *P* = 0.02; ATM levels were measured also in offspring generated by pregnant females injected with saline or VPA at gestation day 12.5 (“sal mice” and “VPA mice”). Sal mice (*n* = 12) vs. VPA mice (*n* = 10), Unpaired *t* test, *P* = 0.02. (**C**) Representative Mecp2 signal and relative quantification from Western blotting experiments performed in *Atm^+/–^* mice (WT vs. *Atm^+/–^*, *t* test: *P* = 0.04; number of samples: WT = 5 vs. *Atm^+/–^* = 8).

In order to unveil the possible involvement of ATM in ASD, we took advantage of 2 different animal models, *Mecp2^y/–^* mice and mice prenatally exposed to valproic acid (VPA), as genetically and pharmacologically linked models of autism. As illustrated in Figure 1A, we found an increased amount of ATM in hippocampi of *Mecp2^y/–^* pups (P6) as well as in hippocampi of young VPA animals (Figure 1B), i.e., mice generated by pregnant dams injected with VPA (600 mg/mL i.p.) at gestation day 12.5. Vice versa, in *Atm^+/–^*, Mecp2 expression was higher with respect to age- and sex-matched WT mice (Figure 1C), suggesting a possible reciprocal correlation between the 2 proteins. Also, whereas *Atm^+/–^* hippocampi display a higher expression of KCC2 (17), in *Mecp2^y/–^* and VPA mouse models of autism, a lower level of KCC2 has been described (26, 28, 30). Thus, starting by these premises, here we investigated the effects of ATM kinase inhibition in vitro and in vivo both in WT mice and animal models of autism.

### ATM kinase inhibitor KU boosts KCC2 expression in vitro and in vivo, anticipates the excitatory-to-inhibitory GABA switch, and potentiates inhibitory neurotransmission.

**Figure 2:**
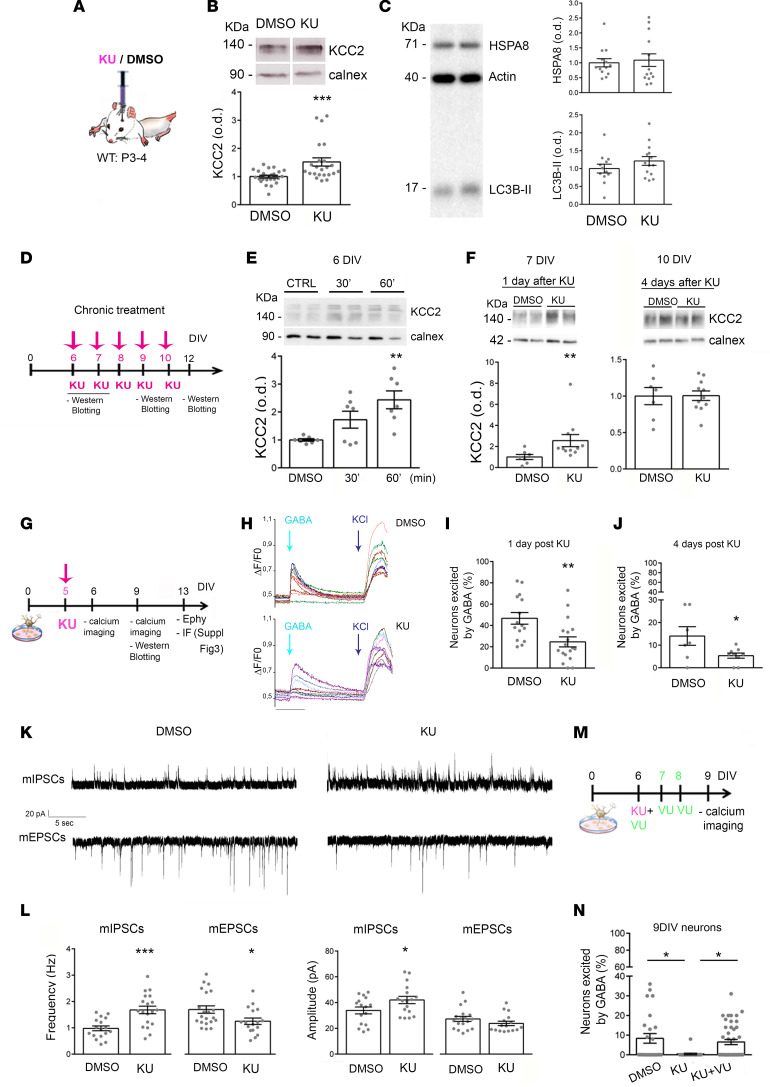
KU regulates GABA development and boosts KCC2 levels in vitro and in vivo. (**A**) In vivo protocol to test KU effects in WT pups and (**B**) measurement of KCC2 levels in brain structures (number of tissues: DMSO = 24 vs. KU = 23; Mann-Whitney *U* test: *P* = 0.0008). (**C**) Quantification of LC3-BII and HSP8A proteins in KU and DMSO mice (LC3-BII, *t* test: *P* = 0.23; number of tissues: DMSO = 12 vs. KU = 14; HSPA8, Mann-Whitney *U* test: *P* = 0.642; number of tissues: DMSO = 13 vs. KU = 14). (**D**) Schematic representation of acute and chronic protocol for the evaluation of KU effects. (**E**) Quantification of KCC2 levels: in the acute protocol (coverslips per group: *n* = 7; 1-way ANOVA followed by Tukey’s multiple comparisons test: *P* < 0.01) or (**F**) 1 day after KU: coverslips DMSO = 7 vs. KU = 11; Mann-Whitney *U* test, *P* = 0.005; 4 days after KU: coverslips DMSO = 7 vs. KU = 11; Mann-Whitney *U* test, *P* = 0.918). (**G**) In the cartoon 5DIV neurons treated once with KU underwent calcium imaging analysis at 6DIV and at 9DIV and electrophysiology and immunofluorescence at 13DIV. (**H**) Traces of calcium transients in 6DIV neurons. (**I** and **J**) Percentage of GABA-responding neurons after KU: 1 day, DMSO vs. KU, *t* test: *P* = 0.004; coverslips: DMSO = 15 vs. KU = 18; total cells: DMSO = 395 vs. KU = 374; independent experiments = 4; 4 days, *t* test: *P* = 0.039; coverslips DMSO = 7 vs. KU = 9; total cells: DMSO = 216 vs. KU = 236; independent experiments = 3. (**K**) Traces of mEPSCs and mIPSCs from 13–14DIV neurons treated with KU at 5DIV and (**L**) analysis of mIPSCs/mEPSCs (mIPSCs: frequency *t* test *P* = 0.0003, DMSO = 17 vs. KU = 19; amplitude: *t* test *P* = 0.0469, DMSO = 16 vs. KU = 17; mEPSCs: frequency *t* test *P* = 0.0262, DMSO = 21 vs. KU = 17; amplitude: Mann-Whitney *U* test *P* = 0.1627, DMSO = 18 vs. KU = 16; independent experiments = 3). (**M**) 6DIV neurons received KU or KU + VU and (**N**) were analyzed for GABA switch: Kruskal-Wallis test followed by Dunn’s multiple comparisons test: DMSO vs. KU: *P* < 0.05; KU vs. KU + VU: *P* < 0.05; DMSO vs. KU + VU (n.s.); fields: DMSO = 25, KU = 21, KU + VU = 44; total cells: DMSO = 204, KU = 163, KU + VU = 334; independent experiments = 4. *P < 0.05, **P < 0.01, ***P < 0.001.

First of all, we asked whether in vivo pharmacological blockade of ATM may affect KCC2 levels. Accordingly with the literature (32, 35, 36), we injected 3 μL of 10 μM KU in the single lateral ventricle of P3–P4 WT mice (Figure 2A) and quantified KCC2 expression 1–2 days later by Western blotting analyses. As shown in Figure 2B, a higher KCC2 expression was found in KU-treated mice. It has been reported that doses higher than 2 μM KU inhibit autophagosome formation (37), so we evaluated the impact of this treatment on basal autophagy in vivo. As a marker of autophagy, we looked at the microtubule-associated protein 1 light chain 3, BII isoform (LC3-BII), in brains explanted from KU-injected pups. Western blotting results indicated that levels of LC3-BII were not affected by KU treatment (Figure 2C). Also, a comparable result was obtained evaluating levels of HSPA8 (Figure 2C), which is a ubiquitous molecular chaperone involved in protein folding and degradation, stress response, endosomal microautophagy, and chaperone-mediated autophagy (38, 39).

In vitro, we evaluated KU effectiveness, duration of action, and toxicity in control primary cultures (see Supplemental Methods and Supplemental Figure 1, A–F; supplemental material available online with this article; https://doi.org/10.1172/jci.insight.133654DS1). We identified the lowest concentration able to produce the desired effects without affecting neuronal health (1 μM KU), and we daily treated hippocampal cultures starting from 6 to 10 days in vitro (DIV) with 1 μM KU (Figure 2D). As described in *Atm^+/–^* hippocampal cultures (17), neurons treated with KU displayed an increased ERK1/2 phosphorylation, suggesting the occurrence of common molecular pathways (Supplemental Figure 1G). In vitro, we detected an increased KCC2 expression 60 minutes after neuronal exposure to the drug (Figure 2E) up to 1 day (Figure 2F), as indicated by Western blotting analysis. Surprisingly, KCC2 levels remained comparable to those found in DMSO/control neurons in the case of (a) long-lasting treatment, i.e., upon a chronic KU administration (Figure 2F); and (b) long and short treatments in mature cells (Supplemental Figure 2H). These data suggested that the higher KCC2 expression triggered by the ATM kinase activity blockade is more likely restricted to the first phase of development whereas it does not occur in mature neurons.

It is known that changes in KCC2 expression affect GABAergic development by shaping the timing of the GABA switch (2, 3, 9, 17, 40). So, we expected to find a modified GABA switch in KU neurons because of the higher KCC2 expression. To address this point, we carried out calcium imaging experiments in cultures loaded with the calcium indicator Fura-2 AM. We treated 5–6DIV neurons with KU (Figure 2G) and evaluated the GABA switch 1 day later. In particular, we measured the number of neurons excited by GABA delivery and the related entity of calcium transients. Imaging experiments clearly indicated that the delivery of exogenous GABA (100 μM) generated a depolarizing response in a lower percentage of cells in the KU condition (Figure 2, H–I). Interestingly, this short application of KU was responsible for a long-lasting effect since the percentage of neurons depolarized by GABA was still reduced 4 days after KU treatment (Figure 2J). Since these results might reflect differences in voltage operated calcium channel (VOCC) expression, we analyzed calcium transients induced by the application of a different depolarizing stimulus, such as 50 mM KCl. We found that KCl was able to generate comparable calcium increases in DMSO controls and KU-treated cultures (Supplemental Figure 1, I–J). This result indicated no differences in terms of VOCC expression in KU neurons with respect to the DMSO controls and confirmed the specificity of GABA switch data upon KU administration.

The short KU treatment during development also affects the basal synaptic activity. We recorded miniature inhibitory and excitatory postsynaptic currents (mIPSCs and mEPSCs) in 14 DIV neurons (Figure 2G) treated with KU at 5–6DIV and found an increased inhibition. Figure 2, K and L, show that inhibitory transmission enhanced both in frequency and in amplitude whereas excitatory events were decreased only in frequency. To demonstrate that these effects directly associate with the higher KCC2 expression, we exploited the specific KCC2 blocker, VU0240551 (VU 1 μM) (41), which does not affect neuronal health (Supplemental Figure 2, A and B). We coincubated WT cultures with KU (at days 5–6) and VU 1 μM (Figure 2M), and we found no differences in the percentage of GABA-responding neurons (Figure 2N). Interestingly, we confirmed these results also in *Atm^+/–^* neurons upon treatment with VU. In fact, premature GABA development (Supplemental Figure 2C) as well as higher I/E ratio (evaluated by recording of mIPSCs and mEPSCs) were fully rescued by VU delivery in *Atm^+/–^* cells (VU treatment: 2, 4, 6, and 8 DIV; electrophysiological recording at 13–14DIV; Supplemental Figure 2D).

### KU counteracts the pharmacologically induced hyperexcitability in neurons.

**Figure 3:**
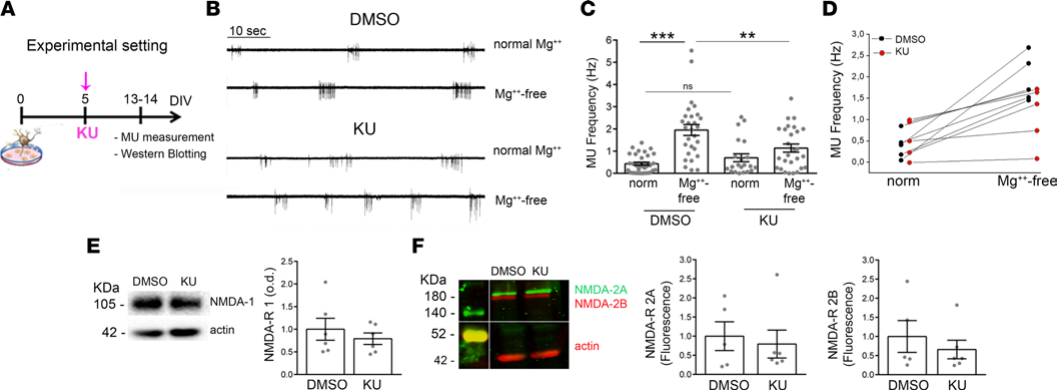
KU-treated cells are more inhibited and less susceptible to hyperactivity. (**A**) Schematic representation of the experimental setting. 13–14DIV neurons, which received KU at 5–6DIV, were tested for the hyperactivity protocol and blotting experiments. (**B**) Traces of multiunit (MU) activity (i.e., spiking activity) evaluated by electrophysiological recordings in the cell-attached modality. Note the increased neuronal firing in mature control neurons upon Mg^2+^ removal from the external solution. Neurons treated with KU at 5–6DIV were resistant to this protocol of hyperexcitability. (**C**) Analysis on the MU mean frequency (Hz) 1-way ANOVA followed by Holm-Šidák multiple comparisons test: DMSO vs. DMSO in Mg^2+^-free sol, *P* < 0.001; DMSO in Mg^2+^-free sol vs. KU in Mg^2+^-free sol: *P* < 0.01; number of independent experiments = 4. DMSO (*n* = 34); DMSO in Mg^2+^-free sol (*n* = 28); KU (*n* = 21); KU in Mg^2+^-free sol (*n* = 26). (**D**) Each dot represents the mean of MU activity analyzed per glass before and after Mg^2+^ removal in the 2 experimental groups (Kolmogorov-Smirnov test < 0.001). (**E** and **F**) Blotting analysis displays no difference in NMDA-Rs’ expression in neurons treated with KU during development (KU treatment: 6–7DIV; Western blotting: 14DIV neurons). NMDA-R-1: DMSO (*n* = 6) vs. KU (*n* = 6); *t* test: *P* = 0.57. NMDA-R-2A: DMSO (*n* = 5) vs. KU (*n* = 6); *t* test: *P* = 0.99. NMDA-R-2B: DMSO (*n* = 5) vs. KU (*n* = 6); *t* test: *P* = 0.64. **P < 0.01, ***P < 0.001.

In a good accordance to the significant enhancement of inhibitory activity found in 13–14DIV neurons treated with KU at 5–6DIV, immunofluorescence analysis also revealed a higher mean intensity and mean size of vesicular GABA transporter–positive puncta and reduced vesicular glutamate transporter 2–positive signal (Supplemental Figure 3, A and B). Moreover, we found that KU-treated cells were also less susceptible to a paradigm of hyperexcitability acutely generated in vitro by exposing neurons to a Mg^2+^-free external medium, i.e., sustaining NMDA receptors’ activation (42, 43). Multiunit (MU) activity, which is known to reflect the spiking activity of principal neurons (44), was recorded by voltage-clamp in the cell-attached modality (Figure 3, A and B). This method allows us to monitor the spiking activity of the recorded neuron as well as of its immediate neighbors (44). As shown in Figure 3C, while the MU number was significantly higher in 14DIV neurons exposed to the Mg^2+^-free medium with respect to normal Krebs-Ringer–HEPES (KRH), no increment in the MU frequency (Figure 3, C and D) was observed after Mg^2+^ removal in neurons treated with KU at 5–6DIV. We excluded that this effect resulted from a reduction of NMDA receptor (NMDA-R) subunits’ expression (NR1, NR2A, and NR2B), as indicated by Western blotting data (Figure 3, E and F).

### KU mediates the rapid Egr4-dependent activation of the Kcc2b promoter and Mecp2 transcription.

**Figure 4:**
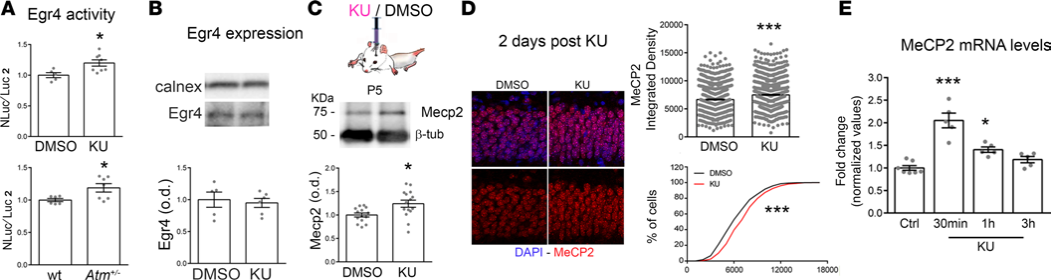
KU triggers Egr4 activation and increases Mecp2 transcription. (**A**) Measurement of luciferase expression in rat cultures after KU treatment (above) and in *Atm^+/–^* cells (below). Increased Egr4 activity on *Kcc2b* promoter has been found in KU cells (NanoLuc/Luc2 normalized values, unpaired *t* test: *P* = 0.02; number of independent experiments = 3; number of samples: DMSO *n* = 5 vs. KU *n* = 8). *Atm^+/–^* cultures display also increased Egr4 activity on *Kcc2b* promoter (NanoLuc/Luc2 normalized values, Unpaired *t* test: WT vs. *Atm^+/–^*: *P* < 0.02; number of independent experiments = 3; number of samples: WT = 6, het = 7). (**B**) No changes occur in terms of Egr4 expression levels upon KU treatment (*t* test: *P* = 0.71; number of coverslips: DMSO = 5 vs. KU = 6). (**C**) Western blotting experiments performed on tissues explanted from DMSO and KU-injected pups: representative Mecp2 signal and relative quantification (DMSO vs. KU-injected brains, *t* test: *P* = 0.01; number of tissues: DMSO = 14 vs. KU = 15). (**D**) Immunohistological experiments for Mecp2 detection (red) at 1 day after KU injection and relative quantification in DAPI-positive neurons (blue). Integrated density of fluorescence for Mecp2 signal: Mann-Whitney *U* test, *P* < 0.0001. Number of animals: DMSO = 4 vs. KU = 4; number of slices: DMSO = 12 vs. KU = 12; number of images quantified per slices: 6. (**E**) Quantification of *Mecp2* mRNA levels by real-time PCR experiments in neurons treated with KU or DMSO at different time points (Kruskal-Wallis followed by Dunn’s multiple comparisons test: control neurons vs. KU 30 minutes, *P* < 0.001; control neurons vs. KU 60 minutes, *P* < 0.05). *P < 0.05, ***P < 0.001.

To investigate the underlying molecular mechanisms, we explored the possibility that the enhanced *Kcc2* transcription upon KU delivery could be mediated through the activation of Egr4 as in a previous report (45). To this purpose we used a construct including the –309/+42 region of the *Kcc2b* mouse promoter, containing exclusively the Egr4 consensus sequence, as previously demonstrated (46, 47), followed by the NanoLuc luciferase gene reporter. We transfected 5DIV control cultures with the construct, we applied KU 1 day later, and we measured the NanoLuc and Luc2 luciferase activity by a Dual-Luciferase Reporter Assay System after 24 hours. We measured an increased luciferase Egr4 activity in KU-treated cultures (Figure 4A, top) in the presence of unchanged Egr4 expression levels (Figure 4B), indicating that the higher KCC2 expression may occur through the rapid Egr4-dependent activation of the *Kcc2b* promoter. Also, 5DIV *Atm^+/–^* cultures displayed a significantly higher Egr4-dependent activity of the reporter gene with respect to age-matched WT cultures as assessed by Dual-Luciferase Reporter Assay System (Figure 4A, bottom), indicating an enhanced Egr4 activity also in the genetic model expressing reduced level of ATM protein.

Then, based on the result that Mecp2 is highly expressed in *Atm*^+/–^ tissues (see Figure 1C), we evaluated Mecp2 levels in brain tissues of WT pups injected with 3 μL of 10 μM KU. Increased levels of Mecp2 signal were detected in KU-injected brains with respect to controls, as assessed either by Western blotting analysis or by confocal analysis (Figure 4, C and D). Quantitative analysis for *Mecp2* mRNA levels by quantitative real-time PCR indicated that *Mecp2* transcription was transiently potentiated 30 and 60 minutes after KU delivery (Figure 4E). Together these results demonstrated that KCC2 expression was finely modulated by ATM kinase activity through Egr4 and Mecp2 pathways.

### KU rescues abnormal GABA switch and functional alterations in Mecp2^y/–^ neurons.

**Figure 5:**
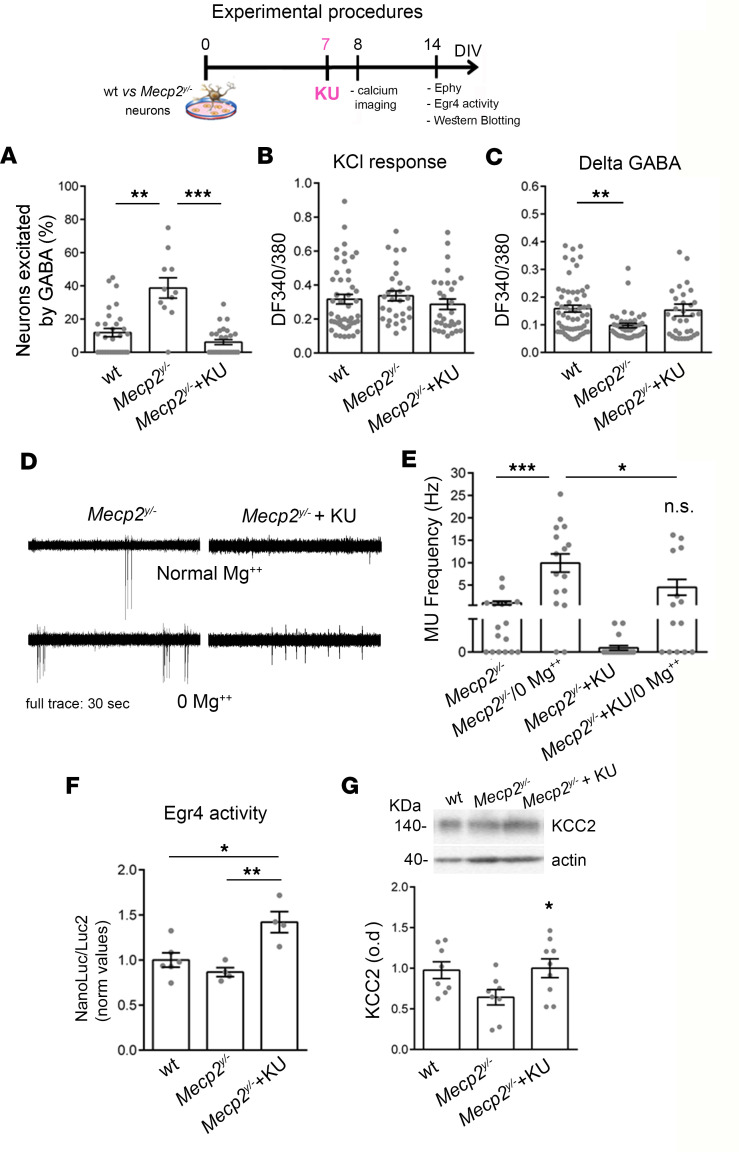
KU effects on *Mecp2^y/–^* developmental and functional alterations. Schematic indicates that 7DIV *Mecp2^y/y^* and *Mecp2^y/–^* neurons were treated with KU (or DMSO) and calcium imaging experiments performed 1 day later and electrophysiology at 14DIV. (**A**) GABA switch experiments performed in 8DIV *Mecp2^y/–^* neurons and WT indicate a rescue in the percentage of GABA-responding cells upon KU delivery (Kruskal-Wallis with Dunn’s multiple comparisons test: WT vs. *Mecp2^y/–^* *P* < 0.01; *Mecp2^y/–^* vs. *Mecp2^y/–^* + KU: *P* < 0.001. Number of fields analyzed WT = 30, *Mecp2^y/–^* = 11, *Mecp2^y/–^* + KU = 28. Four independent experiments. Number of analyzed cells: WT = 227, *Mecp2^y/–^* = 93, *Mecp2^y/–^* + KU = 215). (**B**) *Mecp2^y/–^* neurons treated with KU display a normal response to depolarizing stimuli induced by KCl, suggesting normal VOCC expression (Kruskal-Wallis with Dunn’s multiple comparisons test: *P* = 0.29). (**C**) Calcium transients induced by GABA stimulation indicate lower amount of GABA receptor expression in *Mecp2^y/–^* cells, which is also rescued by KU treatment during development (Kruskal-Wallis with Dunn’s multiple comparisons test WT vs. *Mecp2^y/–^* *P* < 0.01). (**D** and **E**) Cell-attached experiments and quantifications display that *Mecp2^y/–^* + KU neurons are resistant in generating the pharmacological hyperexcitability induced by Mg^2+^ removal. One-way ANOVA with Holm-Šidák multiple comparisons test: *Mecp2^y/–^* vs. *Mecp2^y/–^* in 0 Mg^2+^: *P* < 0.001; 3 independent experiments; number of cells: *Mecp2^y/–^* (*n* = 13), 0 Mg^2+^- *Mecp2^y/–^*, *Mecp2^y/–^*-KU (*n* = 7), 0 Mg^2+^- *Mecp2^y/–^*-KU (*n* = 8). (**F**) KU administration potentiates Egr4 activity on the *Kcc2b* promoter in *Mecp2^y/–^* neurons as indicated by the higher NanoLuc/Luc2 value with respect to the *Mecp2^y/–^* treated with only DMSO (NanoLuc/Luc2 normalized values, ordinary 1-way ANOVA with Tukey’s multiple comparisons test *P* < 0.01; samples (isolated embryos) WT = 6, *Mecp2^y/–^*+ DMSO = 4, *Mecp2^y/–^* + KU = 4). (**G**) KU delivery increases KCC2 expression in *Mecp2^y/–^* neurons as indicated by Western blotting results (1-way ANOVA, *P* = 0.04; samples: WT = 8; *Mecp2^y/–^* = 8; *Mecp2^y/–^* + KU = 9; 3 independent experiments). *P < 0.05, **P < 0.01, ***P < 0.001.

Since *Mecp2^y/–^* pups displayed higher ATM levels (see Figure 1A) and *Mecp2^y/–^* neurons showed an excitatory GABA action and low KCC2 levels (26), we tested the possibility to rescue these defects by KU treatment. A schematic representation about the experimental procedures is shown in Figure 5. Data collected by calcium imaging experiments revealed in 7–8DIV *Mecp2^y/–^* cultures that the higher percentage of GABA-responding neurons was normalized by KU delivery (Figure 5A). Once again, this effect was not linked to increased VOCC expression in the KU group since no differences in the amplitude of KCl-induced calcium responses were detectable among the groups (Figure 5B). Interestingly, in *Mecp2^y/–^* neurons, the reduced calcium peaks induced by GABA delivery suggested a significant alteration in the GABA-A receptor expression, which was restored by KU application (Figure 5C). Also, the neuronal hyperexcitability induced by exposing neurons to a 0 Mg^2+^ external medium was observed only in 14DIV *Mecp2^y/–^* cultures, as indicated by the significantly higher MU frequency with respect to control solution (Figure 5, D and E). No significant differences in terms of firing frequency could be detected in *Mecp2^y/–^* neurons exposed to 0 Mg^2+^ solution treated with KU during development (Figure 5, D and E). Accordingly, *Mecp2^y/–^* cells treated with KU displayed a potentiated Egr4 activity (Figure 5F) and normalized KCC2 levels (Figure 5G).

### KU rescues abnormal GABA switch and autism-like behavior in VPA mice.

**Figure 6:**
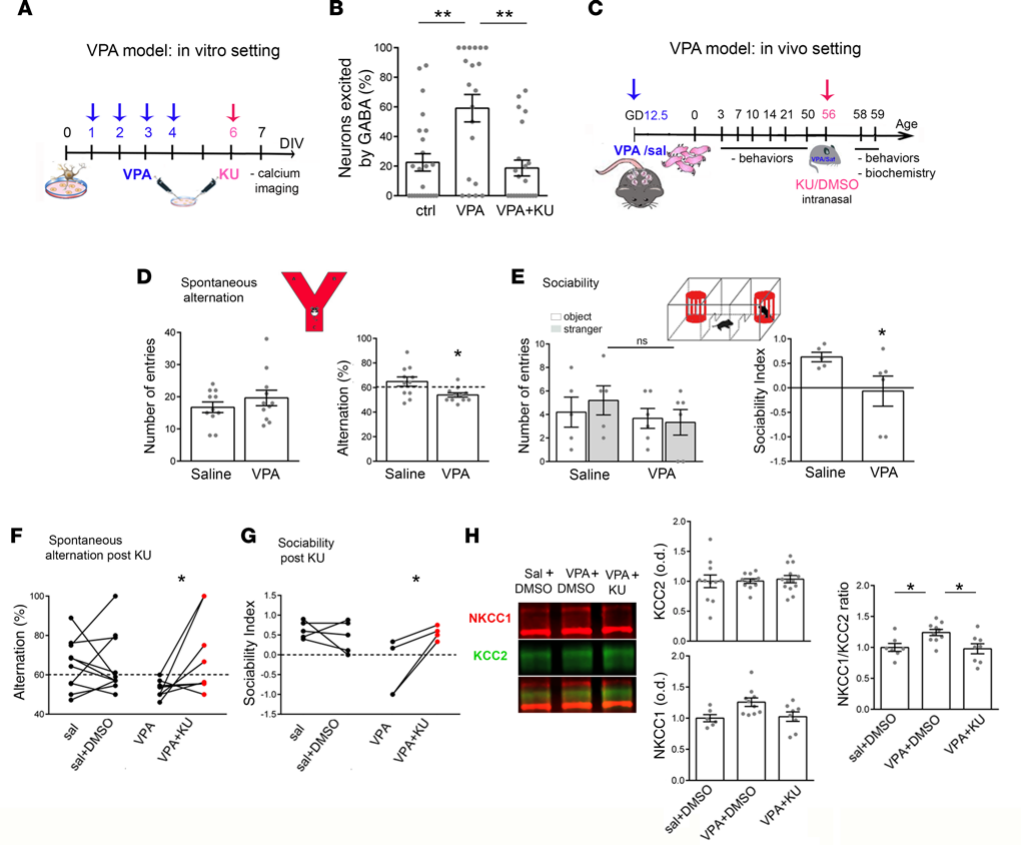
In vitro and in vivo effects of KU in the VPA model of autism. (**A**) The schematic representation displays that WT cells received VPA 2 mM starting from 1DIV to 4DIV as in ref. 30. KU 1 µM was added to 6DIV cultures, and calcium imaging experiments were carried out 1 day later. (**B**) Percentage of GABA-responding neurons exposed to VPA: Kruskal-Wallis followed by Dunn’s multiple comparisons test, ctrl vs. VPA: *P* < 0.01; VPA vs. VPA + KU: *P* < 0.01; number of fields analyzed ctrl = 24, VPA = 20, VPA + KU = 22; number of analyzed cells: ctrl = 237, VPA = 136, VPA + KU = 127. Three independent experiments. (**C**) Scheme of the experimental procedure: pregnant female (gestation day 12.5) received VPA or saline. The generated offspring were monitored in terms of growth delay (at P3, P7, P10, P14, P21), eye opening (P13–P14), olfactory motivation/nest bedding test (P10), and spontaneous alternation and sociability (P50). Adults received KU or DMSO intranasally and 2–3 days later were newly challenged in the spontaneous alternation test and sociability test and eventually sacrificed for biochemistry. (**D**) Spontaneous alteration test (analysis performed in **M**) percentage of alternation, *t* test, *P* = 0.01; number of animals: sal = 11 vs. VPS = 11. (**E**) Sociability test: social index (analysis performed in **F**) *t* test, *P* = 0.03; number of animals: sal = 5 vs. VPS = 6. (**F**) Spontaneous alteration test after KU: percentage of alternation sal vs. sal + DMSO paired *t* test: *P* = 0.92; VPA vs. VPA + KU paired *t* test: *P* = 0.04; number of animals: sal (which then received DMSO, i.e., sal + DMSO) = 10 vs. VPA (which then received KU; i.e., VPA + KU) = 8. (**G**) Sociability Index (SI) after KU: sal vs. sal+DMSO paired *t* test: *P* = 0.47; VPA vs. VPA + KU unpaired t test: *P* = 0.04; number of animals: sal (which then received DMSO, i.e., sal + DMSO) = 5 vs. VPA (which then received KU; i.e., VPA + KU) = 4. (**H**) Western blotting experiments performed on tissues explanted from adult control mice (sal + DMSO), VPA + DMSO, and VPA + KU-treated adult animals: representative NKCC1 and KCC2 signals and relative quantification (1-way ANOVA followed by Tukey’s multiple comparisons test *P* = 0.011; number of animals: sal + DMSO = 7, VPA + DMSO = 10, VPA + KU = 8). *P < 0.05, **P < 0.01.

To address the therapeutic potentiality of KU in the valproate mouse model of autism (VPA model), we first performed in vitro experiments. We treated WT neurons with VPA from 1DIV to 4DIV (see the cartoon in Figure 6A). By calcium imaging experiments we found a higher percentage of neurons that responded to exogenous GABA with a neuronal depolarization. This defect was fully normalized by KU treatment (Figure 6B). Once again, no changes in VOCC expression occurred the in KU group as suggested by the comparable amplitudes of KCl-induced calcium responses among the KU-treated and vehicle-treated VPA cells (Supplemental Figure 4A).

Finally, we moved to in vivo experiments to evaluate KU effects in the VPA mouse model of autism, since these mice display a delayed GABA switch, a long-lasting excitatory action of GABA (29), and higher ATM levels (see Figure 1B). We treated pregnant dams at gestation day 12.5 with VPA (or saline) in order to induce an autism-like phenotype in the generated offspring (VPA mice; see Figure 6C) (48, 49). According to published data, VPA mice displayed a growth delay as indicated by the reduced body weight, delayed eye opening, and important defects in a battery of behaviors (Supplemental Figure 4B) such as communicative behavior, cognitive function, and social performances (48). Communication is generated by the integration of multiple sources of information, such as those triggered by olfaction (50), so we evaluated the olfactory motivation test/nest bedding test in saline and VPA mice. As expected, VPA animals displayed significant impairments in the percentage of arrivals at home cage bedding (Supplemental Figure 4B). Also, VPA mice displayed important defects in cognitive function and social behavior that we evaluated, respectively, by the spontaneous alternation test and the sociability test (Figure 6, D and E). (29). So, we treated young adult VPA mice with KU and assessed its effects via behavior and biochemistry. We delivered KU in VPA mice by the intranasal route (10 mM KU; 7.5 mg/kg; see Figure 6C) and found 2–3 days later the complete restoration of cognitive defects as well as restoration of impairments in sociability (Figure 6, F and G). Since the GABA-inhibitory or -excitatory effects arise from the expression levels of both chloride cotransporters KCC2 and NKCC1, which complementarily regulate intracellular chloride fluxes and GABA direction/response (9), we measured KCC2 and NKCC1 levels in the VPA model. As found for KCC2, NKCC1 expression also appeared marginally altered in the VPA mice, but analysis of the NKCC1/KCC2 ratio, which is more informative in terms of excitatory or inhibitory GABA action, revealed a significant increment in VPA animals that were normalized by the intranasal treatment with KU (Figure 6H).

Notably, we have identified a suitable treatment for autism, but further experiments should be performed to assess whether the beneficial effect on ASD core symptoms is also long-lasting.

Altogether these data strongly indicate that KU treatment is able to ameliorate autistic traits in VPA mice and that the pharmacological tuning of ATM activity could be exploited in ASD treatment.

## Discussion

**Figure 7:**
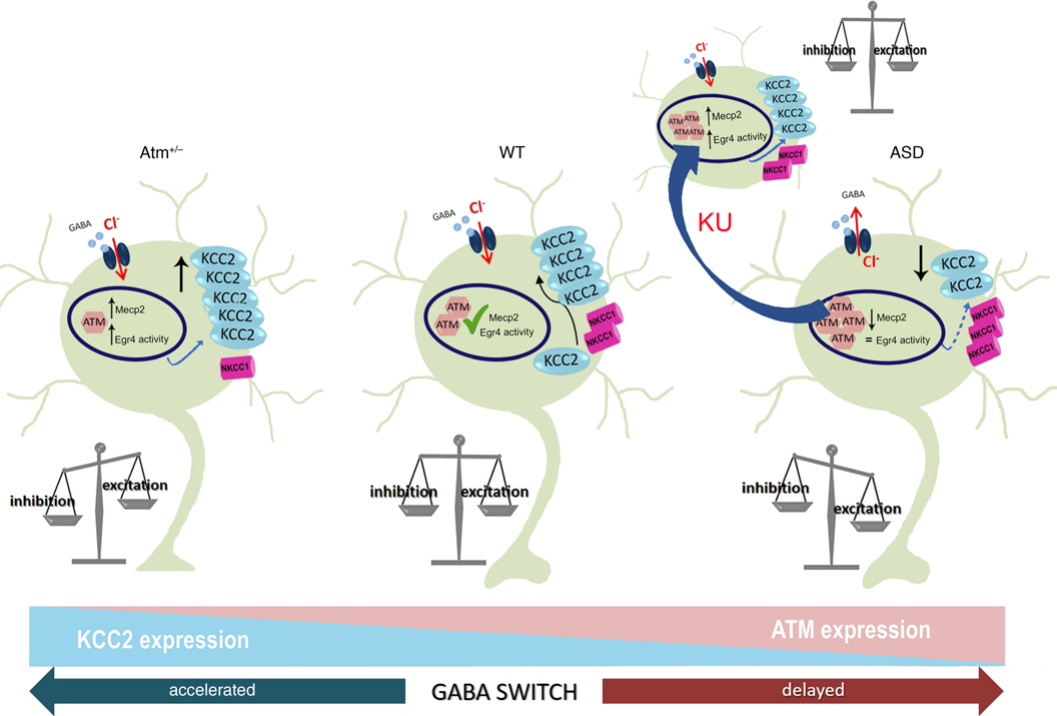
Cartoon of the proposed model. Our model proposes that in WT developing neurons the proper expression and functioning levels of ATM, Mecp2, and Egr4 mediate the right KCC2/NKCC1 amount and thus the correct maturation of GABAergic system. This condition generates the physiological excitatory/inhibitory balance in the network. In *Atm^+/–^* neurons the higher expression of Mecp2 and Egr4 activity generate increased levels of KCC2 associated to reduced NKCC1 and consequently a premature GABA switch. Based on the trophic GABA action, this mediates a higher inhibition. On the contrary, in a condition of ASD the higher ATM levels associates to a lower Mecp2 expression, reduced KCC2 quantity, and increased NKCC1 with a persistent excitatory GABA effect. The transient blockade of ATM kinase action achieved by KU rescues these pathological modifications, leading to the normal neuronal firing.

In this study we demonstrated that the targeting of ATM kinase activity can be exploited to normalize neuronal development and brain function. In particular, we provided the proof of principle that the application of ATM kinase inhibitor KU55933 (KU) generates a higher activation of the transcription factor Egr4 and a higher transcription and expression of the epigenetic regulator Mecp2 determining increased KCC2 levels (see the cartoon in Figure 7). Consequently, it promotes the development of the GABAergic system and, through the trophic action of GABA itself, an increased inhibitory transmission. Therefore, it may be used to treat pathological conditions characterized by KCC2 deficiency (1–3, 51, 52). KU is already used in preclinical studies for cancer treatment (31, 35, 36). It is a small molecule with possible undesirable side effects depending on the dosage and route of administration. We identified the lowest concentration free from toxic effects for the in vitro and in vivo experiments, and by the intranasal delivery we basically exclude systemic effects. Several studies indicate that KU displays a good selectivity for the ATM kinase. In fact, (a) 10 mM KU has no significant effects on unspecific pathways, such as the CREB transcriptional basal activity (53); (b) the dose of 5 μM KU is the highest nontoxic drug concentration linked to a cell viability more than 85% (54); (c) 2 μM KU does not inhibit the cell survival (37); and (d) 2 μM KU inhibits rapamycin-induced autophagosome formation and amino acid starvation-induced autophagic flux in cell lines (37). Since we used 10 μM KU in in vivo experiments, we checked the possible KU inhibition of basal autophagy by measuring levels of autophagy markers such as LC3-BII protein and of endosomal microautophagy and chaperone-mediated autophagy, HSP8A protein. We did not find any changes in these proteins’ expression even if KU concentration applied in vivo was much higher with respect to the one linked to the autophagy inhibition found in vitro. We justify these results by the evidence that (a) in the ventricle KU gets diluted, so the effective concentration is lower with respect to the injected one; (b) starting by the same drug concentration different effects can be induced in different protocols (in vivo vs. in vitro); and (c) the inhibition of autophagosome formation has been proved for KU in an autophagy-activated protocol whereas we evaluated KU effects in unstimulated neurons/mice.

In a previous study we showed a novel role of ATM in the regulation of the development of GABAergic inhibition (17). Here we investigate whether, and to what extent, the ATM kinase activity affects neurotransmission during physiological brain development and in neurodevelopmental disorders. Our in vitro results corroborate the link between ATM activity and KCC2/NKCC1 expression and clarify that among the ATM functions there is the control of NKCC1/KCC2 balance, thus resulting in a potentially new biological substrate to target in developmental disorders affected by NKCC1/KCC2 deregulation. Importantly, here we demonstrate that ATM activity plays an essential role in the maturation of GABAergic system leveraging on 2 transcription factors, Mecp2 and Egr4, which control the expression levels of several proteins among which KCC2 is one. In fact, with a reduced but still present ATM activity (i.e., KU-treated cells/mice and in *Atm*-heterozygous neurons/mice), these 2 factors nicely work, leading to correct KCC2 levels. Vice versa, in Mecp2-null brains, in which we found higher ATM expression, KCC2 was reduced, and KU administration normalized its expression, potentiating Egr4 activity. Thus, variations in ATM levels or activity reflect opposed KCC2 expression and alteration in GABAergic development based on Egr4- and Mecp2-dependent mechanisms.

Our findings acquire particular relevance since cognitive dysfunctions in psychiatric and neurodevelopmental disorders have been recently linked to proteins involved in DNA DSBs machinery (22, 55–57). In particular, it has been demonstrated that neuronal responses to external stimulation are associated to the formation of DSBs (55, 56). Exposure of mice to physiological learning behaviors results in activity-induced DSBs restricted to loci enriched for the early response genes, including *Fos*, *Npas4*, *Egr1*, and *Nr4a1* (22, 23), which may affect synaptic function by epigenetic mechanisms. In this scenario, these studies, including our present results, demonstrate that defective DSB repair factors generate neurological abnormalities and that a better understanding of mechanisms underlying these alterations will be of enormous significance.

Also, several animal models demonstrated that increased excitatory/inhibitory balance occurs in a large case of psychiatric pathologies resulting from genetic modifications, as indicated in (a) *Oxt^–/–^* or *Scn1a^+/–^* animals (the mouse models of myoclonic epilepsy associated with autistic behavior) (5, 58), (b) FMRP mice (genetic animal model of fragile X syndrome/mental retardation) (59), (c) REELER mouse model of schizophrenia (60, 61) (d) *Mecp^y/–^* mice for Rett syndrome (62, 63), and (e) the pharmacologically induced VPA model. Our findings highlight ATM kinase as a new potential target for restoring the proper equilibrium between the glutamatergic and GABAergic afferents in conditions characterized by hyperactivity. Indeed, results collected here in *Mecp2^y/–^* neurons and the VPA model indicate that the ATM tuning positively affects defective neuronal development, leading to a normal GABAergic maturation and function. Finally, ATM signaling has been found consistently elevated in cells derived from Huntington mice and in brain tissues from Huntington mice and patients. Notably, the reduction of ATM expression, obtained by crossing the murine *Atm* heterozygous null allele onto mice expressing full-length human Huntington, ameliorates multiple behavioral deficits and partially improves neuropathology in the Huntington mouse model (64, 65). Also, in 2 mouse models for Huntington disease, the cognitive defects have been demonstrated to be linked to a reduced KCC2 expression that generates a condition of excitatory GABA (65). These results further support our hypothesis of ​​placing ATM among the pathways responsible for the correct development of the central nervous system.

Our data indicate that among the pathological modifications that occurred in the mouse models of autism such as in *Mecp2^y/–^* mice and in the VPA model, a higher ATM activity contributed to the generation of the altered neuronal phenotype. Further experiments are needed to better investigate the involvement of ATM in other neurological states, such as autism and epilepsy, since these pathologies may result from insufficient KCC2 levels and hyperexcitability. In particular, KU application in developmental diseases offers, as a positive example of drug repositioning, the big benefit to shorten time of drug characterization and to develop an old drug in a new field.

## Methods

### Animals

All efforts were made to minimize the number of animals used and their sufferings. Mice were maintained under standard laboratory conditions (room temperature [22 ± 2°C] with 12-hour light/12-hour dark cycle [lights on at 8:00 am] with food and water ad libitum) and kept 5 per cage. Tests were conducted during the light phase of the circadian cycle between 9:00 am and 1:00 pm. *Atm* heterozygous mice were generated crossing *Atm* heterozygous males and C57BL/6 females, *Mecp2*-null mice were provided by Nicoletta Landsberger (BIOMETRA), and pregnant C57BL/6 dams were purchased from Charles River Laboratories.

### Cell cultures

Characterization of KU55933 was conducted on hippocampal neurons established from E18 rat littermates (Charles River Laboratories) as described previously (66). *Atm* genetically modified cultures were established from E18 embryos; *Mecp2* genetically modified neuronal preparations were obtained by P0 pups in order to minimize the number of pregnant females sacrificed (17).

### Genotyping

Genotyping for *Atm* and *Mecp2* animals was performed using PCR techniques. After DNA purification (67), 3 μL of DNA was added to 7.5 μL of master mix (GoTaq Promega), 0.25 μL of each primer, and 3.75 μL of Nuclease-free water for *Atm* genotyping and 7.5 μL master mix (GoTaq Promega), 0.375 μL of each primer, and 3.375 μL of Nuclease-free water for *Mecp2*. The DNA was amplified using a thermocycler (Bio-Rad). Primer sequences for *Atm* genotyping: 5′-GTAGTAACTATTAGTTTCGTGCA-3′, 5′-TAGGGTGTAGTAGTGGAGGA-3′, 5′-ACGTAAACTCGTCTTCAGACCT-3′. Primer sequences for *Mecp2* genotyping: 5′-CCACCCTCCAGTTTGGTTTA-3′ as reverse primer, 5′-ACCTAGCCTGCCTGTACTTT-3′ as forward primer for *Mecp2*-null allele and 5′-GACTGAAGTTACAGATGGTTGTG-3′ as forward primer for WT allele (68).

### Western blotting

Proteins were extracted starting from explanted tissues or scraped neurons using lysis buffer containing 1% sodium dodecyl sulfate (SDS), 62.5 mM Tris-HCl (pH 6.8), and 290 mM sucrose for tissues and sample buffer containing 3% SDS, 115 mM sucrose, 65 mM Tris-HCl (pH 6.8), and 0.1% β-mercaptoethanol for cells. The total protein concentration of the samples was assessed with a protein assay kit (Thermo Fisher Scientific) using a bovine serum albumin–based standard curve. Protein extracts from tissues or neurons were separated by SDS-PAGE electrophoresis and blotted. Homogenates from cortices and/or hippocampi obtained from P4 WT pups (injected or not with KU), P7 *Mecp2^y/–^* vs. WT male mice, *Atm^+/–^* vs. WT, and VPA mice vs. sal mice were analyzed by Western blotting using mouse anti-NKCC1 clone T4 1:1000 (Developmental Studies Hybridoma Bank), rabbit anti-KCC2 1:1000 (MilliporeSigma 07-432), rabbit anti-ATM 1:500 (MilliporeSigma 071286), rabbit anti-Mecp2 1:1000 (MilliporeSigma M9317), rabbit anti-LC3B1/2 1:1000 (Cell Signaling Technology D11), and HSPA8 1:1000 (Cell Signaling Technology D12F2) antibodies. For scraped neurons: mouse anti–p-ERK 1:1000 (MilliporeSigma E7028), rabbit anti-ERK1/2 1:1000 (Cell Signaling Technology 9102), rabbit anti-KCC2 1:1000 (MilliporeSigma 07-432), rabbit anti-Egr4 1:1000 (Abcam ab198197), mouse anti-NMDA-R1 1:500 (Synaptic System 114-011), rabbit anti-NMDA-R-2A: 1:500 (MilliporeSigma 05-901R), mouse anti-NMDA-2B 1:1000 (NeuroMab 75-101), and HRP-conjugated secondary antibody 1:40000 (Jackson ImmunoResearch 115-035-003 and 111-035-003) were used. Immunoreactive bands were detected by using the Pierce ECL Western Blotting Substrate (Thermo Fisher Scientific) and analyzed with ImageJ software (NIH). Rabbit anti-calnexin 1:1000 (MilliporeSigma C4731), mouse anti-actin 1:1000 (MilliporeSigma A4700), or mouse anti–βIII-tubulin 1:2000 (Promega G712A) was used as loading controls.

### Calcium imaging

Hippocampal neurons were loaded with the membrane-permeable fluorescent Ca^2+^ indicator Fura-2 AM (1 μM; MilliporeSigma) for 30 minutes at 37°C, 5% CO_2_; cells were then washed with KRH buffer (NaCl 125 mM, KCl 5 mM, MgSO_4_ 1.2 mM, KH_2_PO_4_ 1.2 mM, CaCl_2_ 2 mM, HEPES 25 mM; d-glucose 6 mM) and placed into the recording chamber of an inverted microscope (Axiovert 100, Zeiss) equipped with a calcium imaging unit and imaged through a 40× objective (Zeiss). Fura-2 AM was excited at 380 nm and at 340 nm through a Polychrom V (TILL Photonics GmbH) controlled by TillVisION software 4.01. Emitted light was acquired at 505 nm at 1 Hz and images collected with a charge-coupled device Imago-QE camera (TILL Photonics GmbH). Calcium transients were addressed by evaluating the fluorescence ratio F340/380. This parameter was recorded in regions of interest corresponding to neuronal cell bodies and analyzed along sequential images to follow temporal changes.

Basically, after a period of basal recording, GABA was administered at 100 μM concentration, and increments in F340/380 ratio (ΔF340/380, which represents calcium transient) were considered if higher than 0.05 units. Transients occurring within 5 seconds after drug administration were considered actual calcium responses. After GABA administration neurons were washed with KRH and allowed to recover for a few minutes, and then 50 mM KCl was administered to identify viable neurons. Neurons responding to depolarization delivery with a ΔF340/380 smaller than 0.1 units were excluded from the analysis.

### In vitro electrophysiology

The ATM kinase inhibitor KU55933 10 mM dissolved in DMSO (used at final concentration of 1 μM) was applied for 4 consecutive days (staring from 7DIV 11DIV neurons) without changing the neuronal medium, and electrophysiological properties were evaluated on 12DIV cells. In the acute treatment protocol, we treated neurons with KU at 5–6DIV and we evaluated activity in 13–14DIV neurons. Excitatory and inhibitory postsynaptic currents in miniature (mEPSCs and mIPSCs) were measured by whole-cell patch clamp procedure using an Axopatch 200A amplifier (Axon Instruments) in the voltage-clamp mode. mEPSCs and mIPSCs were sampled at 10 kHz and filtered at 2–5 kHz. External solution (KRH) consisted of (in mM) 125 NaCl, 5 KCl, 1.2 MgSO_4_, 1.2 KH_2_PO_4_, 2 CaCl_2_, 6 glucose, and 25 HEPES-NaOH (pH 7.4). Postsynaptic events were recorded in the presence of 1 μM tetrodotoxin (Tocris Bioscience). Recording pipettes were pulled from capillary glass (World Precision Instruments) using a 2-stage puller (Narishige) and had tip resistances of 3–5 MOhm when filled with intracellular solution (in mM): 130 Cs-gluconate, 8 CsCl, 2 NaCl, 10 HEPES, 4 EGTA, 4 MgATP, and 0.3 Tris-GTP. Voltage-clamp recordings were performed at holding potentials of –70 mV and +10 mV for mEPSCs and mIPSCs, respectively. Recordings were performed at room temperature. Data were analyzed offline (pClamp-10 software, Axon Instruments). To be taken into account, mEPSCs had to exceed a threshold of 8 pA whereas for mIPSCs it had been set at 6 pA. The I/E ratio was calculated by dividing mIPSCs’ and mEPSCs’ frequencies measured in the same neuron. MU was detected in cell-attached configuration clamping neurons at –50 mV rather than –70 mV, and hyperexcitability was measured by applying KRH external solution with 0 Mg^2+^ during the entire recording session. In this case intracellular solution was (in mM) 130 K-gluconate, 10 KCl, 1 EGTA, 10 HEPES, 2 MgCl_2_, 4 MgATP, and 0.3 Tris-GTP. Regarding data collected using the KCC2 blocker VU0240551, we used the final concentration of 1 μM (starting solution 10 mM in DMSO).

### Luciferase assay

5DIV hippocampal neurons were cotransfected with pNL1.1 (*Nluc*) vector (Promega) containing the –309/+42 region of the *Kcc2* mouse gene and pGL4.54 (*luc2*/TK) vector (Promega) using Lipofectamine 2000 (Invitrogen) according to the manufacturer’s protocol. Then, 48 hours after transfection, cultures were briefly washed with PBS and lysed in Passive Lysis Buffer (Promega). Both *Nluc* and *luc2* luciferase activities were measured using Nano-Glo Dual-Luciferase Assay System (Promega).

pNL1.1 (*Nluc*) vector was modified by introducing in its multiple cloning region the –309/+42 region of *Kcc2* mouse gene containing the Egr4 consensus sequence as the only binding site for transcription factors (as described previously in refs. 46, 47). Subloning procedures were performed by Bio-Fab Research srl (Rome).

### Immunofluorescence

P4 WT C57BL6/J pups were injected with 10 μM KU or vehicle. Then, 24 hours after the injection pups were euthanized, and the brains were removed and fixed in 4% paraformaldehyde for 48 hours. Brains were then included in 4% low melting point agarose (MilliporeSigma) in 1× PBS. After agarose polymerization sections of 50 μm thickness were obtained using a VT1000S vibratome (Leica Microsystems). Immunofluorescence staining was carried out on free-floating sections at the level of dorsal hippocampus. Staining was performed using a primary antibody against Mecp2 (MilliporeSigma) followed by incubation with the specific secondary antibodies rabbit polyclonal antibody anti-Mecp2 (MilliporeSigma M9317) and Alexa Fluor 568 (Invitrogen, Thermo Fisher Scientific, A-11011), counterstained with DAPI, and mounted in Fluorsave (Merck). Images were acquired and analyzed as described (70).

### Quantitative real-time PCR

6DIV WT neurons were treated with KU and homogenized prior to RNA extraction using TRIzol reagent (Invitrogen). Total RNA was isolated using the Direct-zol RNA MiniPrep isolation kit (Zymo Research) according to the manufacturer’s protocol. The RNA was eluted with 25 μL DNase/RNase-free water, quantified using NanoDrop 2000c spectrophotometer (Thermo Fisher Scientific), and optical density 260/280 nm ratios were determined. Reverse transcription was performed using 1 μg RNA with a High Capacity cDNA RT kit (Applied Biosystems). Real-time PCR (qRT-PCR) was performed using a CFX96 thermal cycler (Bio-Rad) in a final volume of 10 μL using SYBR Green technique (SensiFAST SYBR Lo-ROX, Bioline). Mecp2 was analyzed at least in duplicate, and data analysis was performed with the ΔΔCt method and expressed as fold change. *Mecp2* mRNA levels were normalized to GAPDH.

### In vivo KU injection

Four days after the delivery, WT C57BL6/J pups (*n* = 24) were anesthetized by cold-ice procedure. After 5 minutes animals received in the ventricle of the right hemisphere a single unilateral injection of 3 μL of 10 μM KU in DMSO (*n* = 12) or DMSO only (*n* = 12). The day after, cortical and hippocampal tissues were explanted from both the ipsi- and contralateral hemispheres and stored at –20°C. Regarding the intranasal delivery, KU55933 or vehicle (DMSO) was administered to P40 VPA/control mice by intranasal route at a dosage of 7.5 mg/kg. The mice had been previously anesthetized, and the total volume was administered 3 μL at a time, alternating the 2 nostrils.

### VPA treatment

#### In vitro experiments.

Hippocampal neurons were treated with VPA (MilliporeSigma), 2 mM, starting from 1DIV for 4 days. Then, 6DIV neurons received 1 μM KU, and calcium imaging experiments were performed 1 day later.

#### In vivo experiments.

Pregnant WT dams (*n* = 5, 4 months old) received an i.p. injection of VPA 600 mg/kg (MilliporeSigma) at gestation day 12.5, as previously described (71, 72). Control dams (*n* = 4; 4 months old) were treated with saline only. Also, 2 pregnant dams were treated with VPA, but pups were not able to survive.

### Behavioral tests

#### Pups’ appearance.

Mice were weighed at P3, P7, P10, P14, P21, and P50, weighing a random sample of 2–3 perinatal pups rather than the entire litter to prevent perinatal pup loss.

#### Eye opening.

This was checked at P13–P14. Pups were checked for their general appearance at each weighing time point. Numbers of animals are specifically indicated in the legends.

#### Olfactory motivation (nest bedding test).

Saline and VPA mice were tested for olfactory motivation at P10 by placing them in the center of a square (7 cm × 7 cm) plastic tray as previously described (73). One corner of the tray contained bedding from the home cage while the opposite one contained new clean bedding. The home cage bedding had not been changed since the dam was at E18. Mice were placed with their heads pointed toward an empty corner of the tray, forcing it to turn left or right to orientate toward the bedding corners. Mice were allowed up to 1 minute to reach a corner, before the trial was stopped. Trials in which mice failed to move, or to arrive at a bedding corner, were considered uncompleted trials, whereas those in which mice arrived at a bedding corner within 1 minute were considered completed trials. The latency to reach a bedding corner and the type of bedding corner reached were recorded for each of the 3 trials. After each trial the tray was rotated so the beddings were in different orientations relative to the mouse. The nest bedding arrival was calculated as the percentage of trials in which the mice reached the home cage bedding on the total of 3 trials. Number of animals are specifically indicated in the legend.

#### Spontaneous alternation.

Saline and VPA mice were tested before and after intranasal KU delivery (2–3 days later). Spontaneous alternation was measured using a Y-shaped maze constructed with 3 symmetrical gray solid plastic arms at a 120-degree angle (26 cm length, 10 cm width, and 15 cm height) as previously described (74). Mice were individually placed in the center of the maze and were allowed to freely explore the 3 arms for 8 minutes. Arm entry was defined as all 4 limbs within the arm. A triad was defined as a set of 3 arm entries, when each entry was in a different arm of the maze. The maze was cleaned with water and 70% ethanol between sessions to eliminate odor traces. The number of arm entries and the number of triads were recorded in order to calculate the alternation percentage (generated by dividing the number of triads by the number of possible alternations and then multiplying by 100). Number of animals are specifically indicated in the legend.

#### Sociability.

Saline and VPA mice were tested before and after intranasal KU delivery (3 days later). The apparatus was a rectangular, 3-chamber, transparent polycarbonate box as previously described (75). The test mouse was first placed in the middle compartment, and it was allowed to explore all 3 chambers for 10 minutes (habituation). Then, an unfamiliar adult female mouse (never in physical contact with the subject mouse) was placed in an empty wire cage in one side compartment whereas the opposite side contained an empty wire cage. The time spent exploring the unfamiliar mouse and the empty cage was video recorded for 10 minutes. The SI was evaluated as follows: SI = (time exploring the unfamiliar mouse – time exploring the wire empty cage) / (time exploring the unfamiliar mouse + time exploring the wire empty cage). The present task was videotaped and then the parameter scored by an experimenter blind to the treatment offline. Numbers of animals are specifically indicated in the legends.

#### Statistics.

Data were processed by SigmaStat (Systat Software Inc.). The normal distribution of experimental data was assessed using D’Agostino-Pearson normality test. For normally distributed data Student’s 2-tailed *t* test and ordinary 1-way ANOVA with Holm-Šidák multiple comparisons test were performed. Mann-Whitney rank sum test or nonparametric 1-way ANOVA (Kruskal-Wallis test and Dunn’s multiple comparisons test) were applied for non-normally distributed data. Values were expressed as means ± SEM. *P* < 0.05 was considered statistically significant.

#### Study approval.

All the experimental procedures followed the guidelines established by the Italian Council on Animal Care and were approved by the Italian Ministry of Health (Rome, Italy; authorization 991/2016-PR, 369/2019-PR, 210/2017-PR).

## Author contributions

LP performed calcium imaging and Western blotting experiments as well as in vitro electrophysiology and analyzed data, EF performed luciferase measurements and in vivo experiments and analyzed data, CC helped in blotting analysis and immunocytochemistry, LM helped with electrophysiological analysis, LP performed the sociability test, SF and FB helped with KU i.v. injection, GD performed *N*-(ethoxycarbonylmethyl)-6-methoxyquinolinium bromide experiments, NL provided *Mecp2^y/–^* tissues and read the paper, MP and MS read the paper, MM and EM discussed data and read the paper, and FA designed experiments, discussed data, and wrote the paper.

## Supplementary Material

Supplemental data

## Version 1 12/29/2020

In-Press Preview

## Version 2 02/08/2021

Electronic publication
