# Supplementary material for: The DNA repair protein ATM as a target in autism spectrum disorder
Source: JCI Insight. 2021 Feb 8;6(3):e133654. doi: 10.1172/jci.insight.133654 (PMC7934840; doi:10.1172/jci.insight.133654)
Supplement: Supplemental data [file jciinsight-6-133654-s073.pdf]

SUPPLEMENTARY MATERIALS:

Methods:

Western Blotting

Experiments performed in order to analysed KU efficacy have been carried out by exploiting CLx Odyssey (LI-COR Biosciences) and quantified using Odyssey IR Technology by the software Image Studio 2.0.

Immunocytochemistry

After a brief wash in ice-cold phosphate buffer (0.1M PB, pH7.4), neurons were fixed with 4% paraformaldehyde (PFA) in PB for 20 min and specific antibodies for the evaluation of the presynaptic compartment have been used. Specific combinations of the following primary antibodies were used in order to collect images in Suppl. Fig 1 and Suppl. Fig 3: mouse anti-βIII-tubulin 1:400 (Promega), rabbit anti-vGAT 1:1000 (Synaptic Systems), giunea pig anti-vGlut1 1:1000 (Synaptic Systems). Secondary antibodies were conjugated with Alexa-488, Alexa-555, or Alexa-633 fluorophores (Invitrogen, San Diego, CA, USA). Images were acquired using a Zeiss LSM 510 Meta confocal microscope with ×63 objective. Image analysis was performed using the ImageJ software (NIH,Bethesda, MD, USA). For the analysis, vGAT- and vGlut-1-positive puncta per unit length of isolated parent dendrite have been chosen by positive tubulin immunoreactivity. Field per field, isolated dendritic branches have been analyzed in segments of about 30 µm and in total.

Chloride imaging

Intracellular chloride concentrations were evaluated by means of the chloride sensitive dye MQAE (Molecular Probes). Neurons were loaded with 5 µM MQAE for 1 hour at 37°C in culture medium. After washing with KRH solution, cultures were transferred to the recording chamber and acquired on Olympus IX81 inverted microscope, with 20X dry objective (Olympus, UPLFLN NA 0.5). Samples were illuminated with MT20 widefield source and control system with excitation 340 nm and emission filter centred at 500 nm and acquired with Xcellence RT software (Olympus).

SUPPLEMENTARY FIGURES:

Supplementary Figure 1.

A. Representative Western Blotting results display that 1 hour treatment with Etoposide 20 µM produces the ATM dependent DNA damage response as indicated by increased ATM phosphorylation (red) respect to the resting ATM (green), which is prevented in neurons treated with KU55933 10 µM + Etoposide 20 µM. **B**. KU-duration of action in cultured neurons. By the graph is clear that KU effect lasts for at least 24 hours. **C.** We treated hippocampal neurons established from rat embryos with different KU concentrations (1 µM, 5 µM and 10 µM). As indicated here, the acute treatment (4 hours) with 5 µM or 10 µM of KU induces neuronal damage characterized by the appearance of varicosities along tubulin-labelled neuronal processes, already described in [2]. **D.** Long-lasting/chronic treatment (4 days) with 5 µM of KU produces neuronal varicosities and deleterious effects on neuronal survival as indicated by the reduced dendritic arborisation. **E.** Alterations in neuronal calcium homeostasis detected after chronic KU treatment confirm that concentrations higher than 1 µM result in neuronal impairment (Kruskal-Wallis Test followed by Dunn's Multiple Comparison Test, $p<0,01$; total cells analysed: CTRL=244; KU 1µM=221; KU 5µM=200). **F.** Calcium imaging experiments did not indicate changes in Voltage Operated Calcium Channel (VOCC) expression, as indicated by the comparable

calcium transients upon KCl 50 mM stimulation (Mann-Whitney Test, p=0,214; total cells analysed: CTRL=244 vs KU 1μM=219). Note that several studies in literature indicate that concentrations higher than 1 μM KU still display a good selectivity for the ATM kinase further indicating that KU 1 μM is a concentration low enough in order to guarantee both safety, selectivity and effectiveness in neurons [3-6]. **G:** Increased ERK1/2 phosphorylation, 30 and 60 min after acute KU treatment in 7DIV hippocampal neurons. P-ERK/ERK analysis: n=7 samples per group; Kruskal-Wallis Test followed by Dunn's Multiple Comparison Test: ctrl vs KU 30 min p<0,05 and ctrl vs KU 60 min, p<0,01. **H:** Evaluation of KCC2 levels in KU-treated neurons: CTRL (11-12DIV untreated cells) vs 4 days (neurons treated with KU from 6DIV to 10DIV and scraped at 11-12DIV) vs 24 hours (neurons treated once with KU at 12DIV and scraped at 13DIV) vs 60 min (neurons treated with KU at 13DIV and scraped 1 hour later). Kruskal-Wallis Test followed by Dunn's Multiple Comparisons Test, p=0,075. **I-J:** Analysis of calcium transients evoked by a depolarizing stimulus (KCl 50 mM) in control and KU treated cultures was performed in order to exclude the occurrence of possible changes in Voltage Operated Calcium Channels (VOCC) expression and function upon KU delivery, which could reflect variations in neuronal responses to GABA. We found no difference in the amplitude of calcium responses both 1 day and 4 days after KU treatment (**J**), indicating no changes in VOCC expression in KU condition. One-day treatment: Mann-Whitney Test, p=0.145; CTRL n=395; KU=374. Four-days treatment: Mann-Whitney test, p=0.777. CTRL n=216; KU n=236.

SUPPL. FIGURE 2.

A: Chloride-imaging experiments were conducted on neurons treated with VU0240551 (VU) (from 7DIV to 11DIV) loaded with MQAE sensor to confirm the efficacy of the compound in blocking KCC2 co-transporter. Note that reduction in the fluorescence lifetime of the chloride-sensitive dye MQAE measured by means of Fluorescence Lifetime Imaging Microscopy (FLIM) corresponds to increased intracellular chloride concentrations (Student's t-test, p<0,001. CTRL n=138 vs VU n=133). **B:** Calcium imaging experiments indicate that treatment with VU does not affect calcium homeostasis as indicated by the comparable basal calcium levels measured in $Atm^{+/-}$ neurons (het) treated with the KCC2 antagonist. Only weak alterations in calcium homeostasis can be detected in wt neurons upon VU accordingly to what already described in [7] (ΔF340/380 analysis: Kruskal-Wallis Test followed by Dunn's Multiple Comparison Test: p<0,05; number of independent experiments= 5; total cells analysed: wt=362; wt+VU=214; het=342; het+VU=299) **C:** GABA switch in $Atm^{+/-}$ (het) and wt neurons treated or not with VU 1 μM. Note that *Atm* het neurons treated with VU at 2DIV and 4DIV display a rescued % of GABA-responding cells when tested at 6DIV (Kruskal-Wallis Test followed by Dunn's Multiple Comparison Test: wt vs het p<0,01). **D:** I/E ratio evaluated in 13-14DIV *Atm* het vs wt cultures treated with VU 1 μM (treatment at 2-4-6-8DIV). One-way Anova followed by Holm-Sidak's Multiple Comparison Test: wt vs het: p=0<0,01 and het vs het+VU: p<0,01; number of independent experiments=3; number of recorded neurons: wt=15; wt+VU=11; het=11; het+VU=11.

SUPPL. FIGURE 3.

A-B. Immunofluorescence experiments and analysis revealed a higher density of vGAT-positive puncta and a normal density of the vGlut1-positive signal in 14DIV hippocampal neurons exposed to KU during development (6-7 DIV). v-GAT positive puncta mean density (normalized value): Student's t-test, p=0,0310; vGlut positive puncta mean density (normalized value) : Student's t-test, p=0,74. N=2 experiments, n=2 coverslips for each experiment. Scale bar=2,5 µm.

SUPPL. FIGURE 4.

A: Calcium imaging data indicate no changes in VOCC expression, as indicated by comparable calcium transients induced by KCl 50 mM, and in GABA responses in KU-treated and vehicle-treated VPA-cells vs ctrl (KCl response: Kruskal-Wallis followed by Dunn's Multiple Comparisons Test, p=0,47; GABA response: Kruskal-Wallis followed by Dunn's Multiple Comparisons Test, p=0,0678; number of independent experiments=3; total cells analysed: ctrl=237, VPA=136; VPA+KU=127). **B:** Behavioural performances in VPA-mice and saline-counterparts; note that, depending on the test, males (M), females (F) or both display specific features of autistic-like phenotype: **grow delay** (analysis performed on M+F): number of animals: (P3) sal=6 vs VPA=6 ; (P7) sal=8 vs VPA=11; (P10) sal=8 vs VPA=11; (P14) sal=8 vs VPA=8; (P21) sal=8 vs VPA=8; (P55) sal=4 vs VPA=5; Holm-Sidak's Multiple Comparison t-test: p<0,05 for all groups; **Nest Bedding test** (analysis performed on M+F): % of arrivals: sal =88,89±4,73 vs VPA =62,12±6,68; t-test, p=0,009, number of animals: sal=12 vs VPS=22; Time to reach the nest (sec): sal=11,77±1,28 vs VPA=18,54±3,07; t-test, p=0,32; number of animals: sal=12 vs VPS=20; **Eye Opening** (analysis performed on M+F): % sal=89,13±6,25 vs VPA=60±6,5; t-test, p=0,001; number of animals: sal=23 vs VPS=40.

Supplementary materials bibliography

1. Corradini, I., et al., *Maternal Immune Activation Delays Excitatory-to-Inhibitory Gamma-Aminobutyric Acid Switch in Offspring.* Biol Psychiatry, 2018. **83**(8): p. 680-691.
2. Borghesani, P.R., et al., *Abnormal development of Purkinje cells and lymphocytes in Atm mutant mice.* Proc Natl Acad Sci U S A, 2000. **97**(7): p. 3336-41.
3. Addington, A.M., et al., *GAD1 (2q31.1), which encodes glutamic acid decarboxylase (GAD67), is associated with childhood-onset schizophrenia and cortical gray matter volume loss.* Mol Psychiatry, 2005. **10**(6): p. 581-8.
4. Farkas, T., M. Daugaard, and M. Jaattela, *Identification of small molecule inhibitors of phosphatidylinositol 3-kinase and autophagy.* J Biol Chem, 2011. **286**(45): p. 38904-12.
5. Fernandes, N.D., Y. Sun, and B.D. Price, *Activation of the kinase activity of ATM by retinoic acid is required for CREB-dependent differentiation of neuroblastoma cells.* J Biol Chem, 2007. **282**(22): p. 16577-84.
6. Chwastek, J., D. Jantas, and W. Lason, *The ATM kinase inhibitor KU-55933 provides neuroprotection against hydrogen peroxide-induced cell damage via a gammaH2AX/p-p53/caspase-3-independent mechanism: Inhibition of calpain and cathepsin D.* Int J Biochem Cell Biol, 2017. **87**: p. 38-53.
7. Sivakumaran, S., et al., *Selective inhibition of KCC2 leads to hyperexcitability and epileptiform discharges in hippocampal slices and in vivo.* J Neurosci, 2015. **35**(21): p. 8291-6.

Supplementary Figure 1

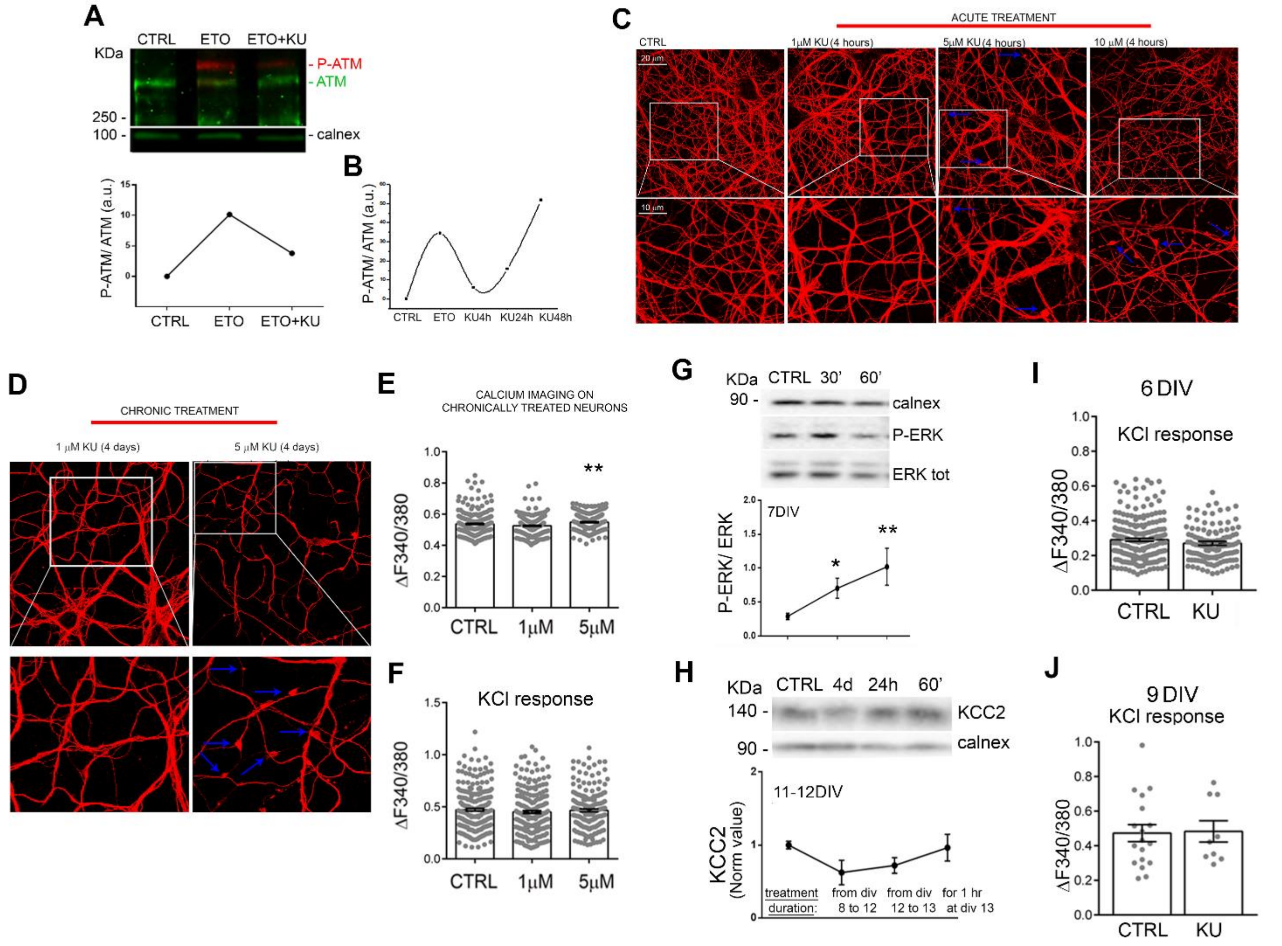

Supplementary Figure 1. A. Representative Western Blotting results display that 1 hour treatment with Etoposide 20 μM produces the ATM dependent DNA damage response as indicated by increased ATM phosphorylation (red) respect to the resting ATM (green), which is prevented in neurons treated with KU55933 10 μM + Etoposide 20 μM. **B**. KU-duration of action in cultured neurons. By the graph is clear that KU effect lasts for at least 24 hours. **C.** We treated hippocampal neurons established from rat embryos with different KU concentrations (1 μM, 5 μM and 10 μM). As indicated here, the acute treatment (4 hours) with 5 μM or 10 μM of KU induces neuronal damage characterized by the appearance of varicosities along tubulin-labelled neuronal processes, already described in [2]. **D.** Long-lasting/chronic treatment (4 days) with 5 μM of KU produces neuronal varicosities and deleterious effects on neuronal survival as indicated by the reduced dendritic arborisation. **E.** Alterations in neuronal calcium homeostasis detected after chronic KU treatment confirm that concentrations higher than 1 μM result in neuronal impairment (Kruskal-Wallis Test followed by Dunn's Multiple Comparison Test, p<0,01; total cells analysed: CTRL=244; KU 1μM=221; KU 5μM=200). **F.** Calcium imaging experiments did not indicate changes in Voltage Operated Calcium Channel (VOCC) expression, as indicated by the comparable calcium transients upon KCl 50 mM stimulation (Mann-Whitney Test, p=0,214; total cells analysed: CTRL=244 vs KU 1μM=219). Note that several studies in literature indicate that concentrations higher than 1 μM KU still display a good selectivity for the ATM kinase further indicating that KU 1 μM is a concentration low enough in order to guarantee both safety, selectivity and effectiveness in neurons [3-6]. **G:** Increased ERK1/2 phosphorylation, 30 and 60 min after acute KU treatment in 7DIV hippocampal neurons. P-ERK/ERK analysis: n=7 samples per group; Kruskal-Wallis Test followed by Dunn's Multiple Comparison Test: ctrl vs KU 30 min p<0,05 and ctrl vs KU 60 min, p<0,01. **H:** Evaluation of KCC2 levels in KU-treated neurons: CTRL (11-12DIV untreated cells) vs 4 days (neurons treated with KU from 6DIV to

10DIV and scraped at 11-12DIV) vs 24 hours (neurons treated once with KU at 12DIV and scraped at 13DIV) vs 60 min (neurons treated with KU at 13DIV and scraped 1 hour later). Kruskal-Wallis Test followed by Dunn's Multiple Comparisons Test, p=0,075. **I-J:** Analysis of calcium transients evoked by a depolarizing stimulus (KCl 50 mM) in control and KU treated cultures was performed in order to exclude the occurrence of possible changes in Voltage Operated Calcium Channels (VOCC) expression and function upon KU delivery, which could reflect variations in neuronal responses to GABA. We found no difference in the amplitude of calcium responses both 1 day and 4 days after KU treatment (**J**), indicating no changes in VOCC expression in KU condition. One-day treatment: Mann-Whitney Test, p=0.145; CTRL n=203; KU=123. Four-days treatment: Mann-Whitney test, p=0.777. CTRL n=18; KU n=9.

Supplementary Figure 2

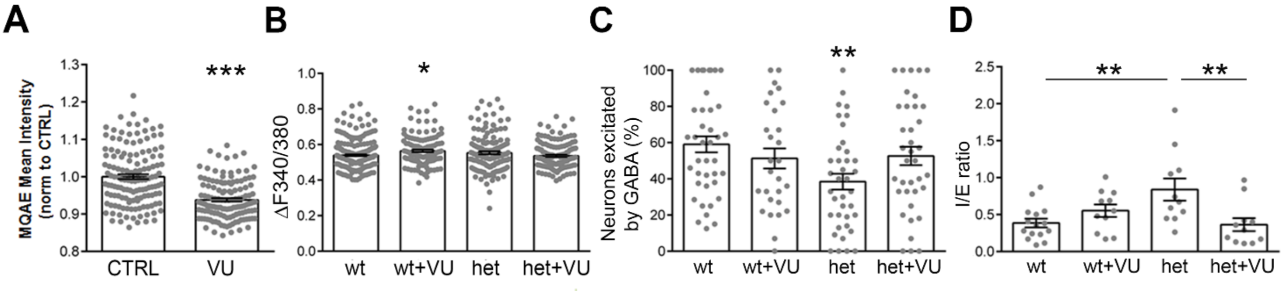

Supplementary Figure 2. A: Chloride-imaging experiments were conducted on neurons treated with VU0240551 (VU) (from 7DIV to 11DIV) loaded with MQAE sensor to confirm the efficacy of the compound in blocking KCC2 co-transporter. Note that reduction in the fluorescence lifetime of the chloride-sensitive dye MQAE measured by means of Fluorescence Lifetime Imaging Microscopy (FLIM) corresponds to increased intracellular chloride concentrations (Student's t-test, p<0,001. CTRL n=138 vs VU n=133). **B:** Calcium imaging experiments indicate that treatment with VU does not affect calcium homeostasis as indicated by the comparable basal calcium levels measured in *Atm*$^{+/-}$ neurons (het) treated with the KCC2 antagonist. Only weak alterations in calcium homeostasis can be detected in wt neurons upon VU accordingly to what already described in [7] (ΔF340/380 analysis: Kruskal-Wallis Test followed by Dunn's Multiple Comparison Test: p<0,05; number of independent experiments= 5; total cells analysed: wt=362; wt+VU=214; het=342; het+VU=299) **C:** GABA switch in *Atm*$^{+/-}$ (het) and wt neurons treated or not with VU 1 µM. Note that *Atm* het neurons treated with VU at 2DIV and 4DIV display a rescued % of GABA-responding cells when tested at 6DIV (Kruskal-Wallis Test followed by Dunn's Multiple Comparison Test: wt vs het p<0,01). **D:** I/E ratio evaluated in 13-14DIV *Atm* het vs wt cultures treated with VU 1 µM (treatment at 2-4-6-8DIV). One-way Anova followed by Holm-Sidak's Multiple Comparison Test: wt vs het: p=0<0,01 and het vs het+VU: p<0,01; number of independent experiments=3; number of recorded neurons: wt=15; wt+VU=11; het=11; het+VU=11.

Supplementary Figure 3

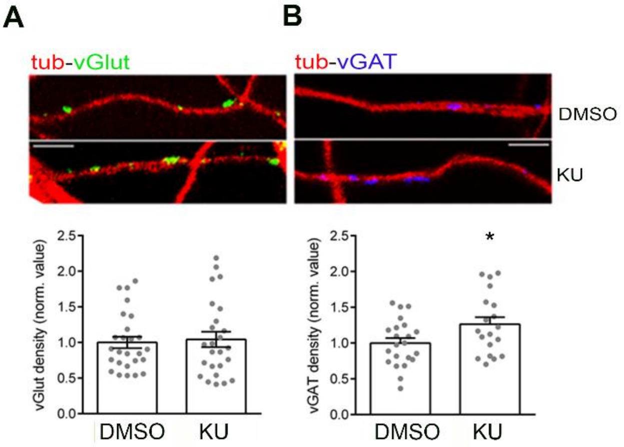

Supplementary Figure 3. A-B. Immunofluorescence experiments and analysis revealed a higher density of vGAT-positive puncta and a normal density of the vGlut1-positive signal in 14DIV hippocampal neurons exposed to KU during development (6-7 DIV). <u>v-GAT positive puncta</u> mean density (normalized value): Student's t-test, p=0,0310; <u>vGlut positive puncta</u> mean density (normalized value) : Student's t-test, p=0,74. N=2 experiments, n=2 coverslips for each experiment. Scale bar=2,5 µm.

Supplementary Figure 4

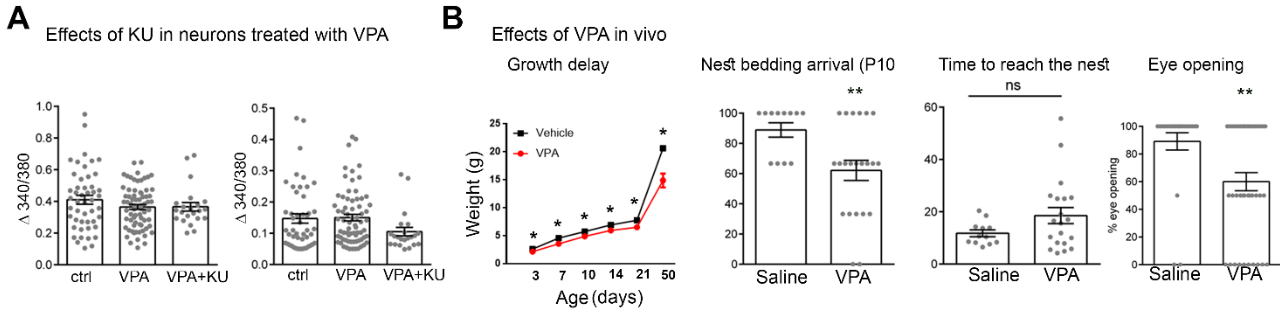

Supplementary Figure 4. A: Calcium imaging data indicate no changes in VOCC expression, as indicated by comparable calcium transients induced by KCl 50 mM, and in GABA responses in KU-treated and vehicle-treated VPA-cells vs ctrl (KCl response: Kruskal-Wallis followed by Dunn's Multiple Comparisons Test, p=0,47; GABA response: Kruskal-Wallis followed by Dunn's Multiple Comparisons Test, p=0,0678; number of independent experiments=3; total cells analysed: ctrl=237, VPA=136; VPA+KU=127). **B:** Behavioural performances in VPA-mice and saline-counterparts; note that, depending on the test, males (M), females (F) or both display specific features of autistic-like phenotype: **<u>grow delay</u>** (analysis performed on M+F): number of animals: (P3) sal=6 vs VPA=6 ; (P7) sal=8 vs VPA=11; (P10) sal=8 vs VPA=11; (P14) sal=8 vs VPA=8; (P21) sal=8 vs VPA=8; (P50) sal=4 vs VPA=5; Holm-Sidak's Multiple Comparison t-test: p<0,05 for all groups; **<u>Nest Bedding test</u>** (analysis performed on M+F): % of arrivals: sal =88,89±4,73 vs VPA =62,12±6,68; t-test, p=0,009, number of animals: sal=12 vs VPA=22; Time to reach the nest (sec): sal=11,77±1,28 vs VPA=18,54±3,07; t-test, p=0,32; number of animals: sal=12 vs VPS=20; **<u>Eye Opening</u>** (analysis performed on M+F): % sal=89,13±6,25 vs VPA=60±6,5; t-test, p=0,001; number of animals: sal=23 vs VPA=40.
